# Fevers

**Published:** 1905-12-02

**Authors:** 


### FEVERS.

**Influenza.**—The derivation of the word influenza, the Italian form of our own word influence, a flowing in or inundation, recalls some of the most striking features of this epidemic disease—features well brought out in a recent discussion of the subject by the Hunterian Society, in which Dr. Clifford Allbutt, Dr. Franklin Parsons,[1] and others took part. In opening the discussion, the former showed how, in almost every century since the twelfth, epidemics of the disease have swept over the world as a flood from the wilds of Northern Central Asia, where it appears to be endemic. Particularly was this the case in the great modern epidemic of 1889-90 when it travelled to Russia along the three great trade routes, was scattered over Europe and England as fast as express trains could carry it, and appeared in New York eight or nine days later, as soon, that is, as steamers could make the passage. In the '89 epidemic in St. Petersburg, people were struck down at the rate of 40,000 a day, while in London 25 per cent. or more of the population suffered. The rapidity and extent of development of an epidemic is accounted for by the short incubation period of the disease—rarely more than forty-eight hours, sometimes less than twenty-four hours, by a widespread susceptibility, and a rapidly developed contagion. Infection appears to be propagated in the sputum and spray from the respiratory tract, and patients without respiratory infection are never, in Allbutt's experience, infectious. Experiments have shown that bacilli from the mouth may be carried by a speaker to a distance of forty feet, and by coughing and sneezing infective matter from the lungs is diffused as spray; hence the Pfeiffer bacillus may probably be conveyed by such acts some feet through the air, and healthy people thus become infected at a considerable "striking distance," as Parsons calls it, from the patient. While infection undoubtedly is from person to person, in the majority of cases it is probably also for a short period conveyed by clothes and other articles. That fomites retain their infectivity for a very short time only seems clear from the investigations of Bulloch [2] who has failed repeatedly, as others have, to grow Pfeiffer's hæmophilic microbe apart from hæmoglobin and has found it difficult to preserve cultures alive. Since the organisms are readily killed by drying, and will only grow between temperatures of 27° and 42° C., it is evident they cannot be carried a great distance from their host or multiply outside his body. In Bulloch's experience, Pfeiffer's bacillus is found now much more rarely than in the early nineties, and he considers that a number of catarrhal diseases due to the micrococcus catarrhalis and other allied cocci frequently but incorrectly go by the name of "influenza," that refuge of the destitute diagnostician. Possibly, as Allbutt suggests, the baccillus influenzæ forms part of the habitual "flora" of the mouth, but we are quite in the dark as to its habits, its means of survival, its home, the circumstances which favour its periodical phases of virulence, and its relation to animal hosts other than man. The protection afforded by an attack is usually very short, about five or six months, and hence a susceptible population is quickly reproduced, but this fact alone will not explain epidemics of influenza. There is no marked seasonal prominence though outbreaks rarely start in summer and are most fatal when the temperature is below the average for the season, but this may be because of a generally lowered vitality. The leisured classes seem to suffer more than the working population, and in proportion to the number of elderly people they contain, and Parsons thinks that there may be something inimical to influenza in the prevalence of conditions which induce filth diseases such as diarrhœa and enteric fever. Glover Lyon [3] thinks that the type of the disease has changed and that the nervous and gastro-intestinal types are now prominent, due respectively to the high pressure existence and restaurant-eating habits of modern men and women. Dealing with some of the clinical aspects of the disease, Allbutt points out that if influenza causes a lobar pneumonia it is almost always associated with lobular complications and attacks both lungs. The sputum is not rusty. Influenza may excavate like tubercle. Pleural and pericardial effusions, and especially empyemas of influenzal origin, do badly. Though showing no striking anatomical change, the heart is in great peril in this fever and no patient who has recently suffered from influenza should be given chloroform. Humphreys has noticed sudden acute dilatation on exertion, and although Allbutt has not met with sudden death as in diphtheria, he points out as a noteworthy physical sign that the cardiac dulness may be found greatly extended in all diameters, reaching from the left mid-clavicular line to beyond the right sternal edge, and rising above to the second rib. The pulse often becomes irregular and may fall to thirty-five or in other cases rise to 140 beats a minute. Typical angina pectoris may occur, due, as Allbutt thinks, to aortitis. Influenza may account for prolonged, puzzling febrile attacks, and be followed for months by profuse paroxysmal sweats. Convalescence is usually very slow and protracted and may then be interrupted by a sudden and complete recovery. Recovery may be as brusque as the onset, and on a sudden, almost fulminating, onset as being extremely typical of influenza Allbutt lays great stress. This suddenness of onset is useful in distinguishing the disease from typhoid fever, with which it may for a time be confounded. The mildest attacks may be followed by severe and protracted depression, but the suicidal cases, due to profound nerve poisoning, seen ten years ago, are less common to-day. In treatment, bed at first and a simple non-nitrogenous diet later are of great importance in minimising the dreary depressing period of convalescence.

[1] Brit. Med. Jour., May 6. [2] Ibid., May 13, p. 1044. [3] Ibid.

(*To be continued.*)

